# Biodegradation of isoproturon by *Pseudoxanthomonas* sp. isolated from herbicide-treated wheat fields of Tarai agro-ecosystem, Pantnagar

**DOI:** 10.1007/s13205-016-0505-8

**Published:** 2016-09-02

**Authors:** Krishna Giri, Shailseh Pandey, Rajesh Kumar, J. P. N. Rai

**Affiliations:** 1 Rain Forest Research Institute, Jorhat, Assam 785 001 India; 2 G. B. Pant University of Agriculture and Technology, Pantnagar, 263145 India

**Keywords:** Phenylurea, Herbicide, Isoproturon, Biodegradation, *Pseudoxanthomonas*

## Abstract

A gram-negative, rod-shaped, isoproturon (IPU) utilizing bacterium was isolated from herbicide-applied wheat fields of Tarai agro-ecosystem, Pantnagar. The phylogenetic sequence analysis based on 16S rRNA sequence revealed that the isolate could be a distinct species within the genus *Pseudomonas.* The isolate was a close relative of *Pseudoxanthomonas japonensis* (95 % similarity) and designated as K2. The bacterial isolate showed positive reaction for oxidase, catalase, and 20 carbohydrates using KB009 Part A and B HiCarbohydrate™ Kit. Degradation experiments were conducted using 200 mg l^−1^ initial IPU as a source of carbon at different pH and temperatures. Maximum IPU degradation by K2 was observed at pH 7.0 and 30 °C, while least degradation at 6.5 pH and 25 °C. Addition of dextrose along with IPU as an auxiliary carbon source increased IPU degradation by 4.72 %, as compared to the IPU degradation without dextrose under optimum conditions. 4-isopropylaniline was detected as a degradation by-product in the medium. The present study demonstrated the IPU metabolizing capacity of a novel bacterial isolate K2 that can be a better choice for the remediation of IPU-contaminated sites.

## Introduction

Isoproturon is an extensively used phenylurea herbicides for control of pre- or post-emergence broad-leaved weeds in cotton, fruits and cereal production. (Sorensen et al. 2003). Ecotoxicological data indicate that IPU and its by-products are harmful to aquatic environments (Widenfalk et al. 2008; Vallotton et al. 2009), and carcinogenic to humans and animals (Hoshiya et al. 1993; Behera and Bhunya 1990). Moreover, extensive use of IPU has been considered as a severe water pollution threat near agricultural catchments in various parts of the world, exceeding the 0.1 µg l^−1^ limits of European Commission (Spliid and Koppen 1998; Muller et al. 2002). Therefore, the use of IPU has been banned or severely restricted in some countries since 2003; however, it is still being used as a potent herbicide in several countries, including India.

Microbial degradation of pesticides in the environment is a reliable, cost effective and eco-friendly remediation technique (Hussain et al. 2011). A variety of IPU degrading soil micro-flora has been recently isolated from contaminated sites in various parts of the world (Badawi et al. 2009; Hussain et al. 2009; Sun et al. 2009). Fortunately, Sphingomonas sp., *Methylopila* sp., *Sphingobium* sp. and *Pseudomonas aeruginosa* strain JS-11 were found promising for complete IPU mineralization (Sorensen et al. 2001; El-Sebai et al. 2004; Sun et al. 2009; Dwivedi et al. 2011). Interestingly, addition of auxiliary carbon source in the medium or pesticide contaminated sites can stimulate microbial growth and significantly enhance the degradation rates of complex organic pollutants (Scow and Hicks 2005). Biostimulation involves the addition of adequate nutrients in the medium to enhance pesticide degradation potential of native microbes (Couto et al. 2010) or to promote cometabolism (De Lorenzo 2008). Biostimulation is one of the major strategies, which has been implemented for the bioremediation of a wide variety of xenobiotics (Kadian et al. 2008; Giri et al. 2015). Although the microbial communities existing in contaminated sites harbour the pesticide catabolising genes and enzymes, lack of electron acceptors or donors, nutrient availability or delayed stimulation of the metabolic pathways essential for degradation acts as limiting factors for pesticide degradation. Under such circumstances, addition of supplementary nutrients or readily available carbon source enhances the degradation of the toxic materials (Cosgrove et al. 2010; Kanissery and Sims 2011).

Uttarakhand, the 27th state of India, is an agrarian state with three-fourth of its total population depending on agriculture. Tarai agro-ecosystem is the major food production zone of Uttarakhand; therefore, pesticide application is also high in this region. Heavy pesticide application poses deleterious effect on non-target beneficial microorganisms, humans and other life forms. Further, ground and surface water contamination, and development of pesticide resistance are some major risks associated with pesticide use. An official survey conducted by Government of India in 2005–06 and 2009–10 indicates that IPU is among the most consumed pesticide in the country (http://www.pesticideinfo.org, retrieved on 24.06.2015 at 9.55 am).

In view of the facts described above, the present investigation aimed to test the IPU degradation potential of a native bacterial isolate that was recovered from IPU-contaminated field.

## Materials and methods

### Chemicals and reagents

Analytical grade IPU and its major metabolites were procured from Sigma Aldrich, USA. All other essential chemicals and microbiological media used in various experiments were of analytical grade and purchased from Himedia Laboratories, India.

### Soil sample collection

IPU-treated composite soil samples at 0–15 cm depth were collected from Norman E. Borlaug Crop Research Centre, Pantnagar, India (29.0210°N, 79.4897°E and 344 m (msl). The soil samples were kept in a refrigerator at 4 °C till bacterial isolations.

### Isolation and identification of IPU degrading bacteria

IPU degrading bacterium was isolated using enrichment culture method, where concentration of IPU was gradually increased from 50 to 200 mg l^−1^ in 100 ml mineral salt broth medium. The flasks were incubated in an orbital shaker at 150 revolutions per minute (rpm) and 30 °C. After 5 subcultures, 1.0 ml culture was inoculated in mineral salt agar plates spiked with 200 mg l^−1^ IPU for the isolation of bacteria. After an incubation of 72 h at 30 °C, bacterial colonies were appeared on the Petri plates. Single colonies were picked and mass multiplied for degradation studies. The isolate was designated as K2 and identified on the basis of morphological, biochemical and 16S rRNA partial gene sequence analysis.

### Determination of minimum inhibitory concentration (MIC)

MIC for bacterial growth was determined using 50, 100, 150, 200, 300, 350 and 400 mg l^−1^ IPU containing mineral salt agar plates after 72 h of incubation at 30 °C.

### Biodegradation of IPU

Biodegradation of IPU was studied at three different pH (6.5, 7.0, 7.5) and temperatures (25, 30, 35 °C), for 20 days. The flasks containing mineral salt medium and 200 mg l^−1^ IPU were inoculated with 1 ml of bacterial culture (OD_600_ = 1.0). The inoculated flasks were then incubated in an orbital shaker at desired temperature and at 150 rpm. Samples were withdrawn aseptically after 5, 10, 15 and 20 days of incubation for determination of residual IPU and its metabolites in the medium.

IPU mineralization potential of bacterial isolate was further assessed by adding 0.5 g l^−1^ dextrose as an auxiliary carbon source in the medium. The flasks were incubated under optimized conditions, i.e., 7.0 pH and 30 °C for 20 days at 150 rpm. Samples were withdrawn after 5, 10, 15 and 20 days incubation to determine the residual IPU and its metabolites in the medium.

Residual IPU and its metabolites were extracted and processed using the previously described methodology (Giri et al. 2015). The IPU extracts were then analyzed using Dionex HPLC equipped with auto sampler and Acclaim 120, C_18_ 5 µm 4.6 × 250 mm column. Samples were eluted using acetonitrile and water (75:25 v/v) at a flow rate of 1 ml min^−1^. The solutes were detected using UV detector at 243 nm. The HPLC analysis was performed at room temperature under isocratic conditions in the Department of Fishery Biology, G.B. Pant University of Agriculture and Technology, Pantnagar, India.

### Statistical analysis

Experimental data were analyzed using MS Excel worksheet for calculation of means and standard errors. Analysis of variance ANOVA was carried out using SPSS 16 statistical software.

## Results and discussion

### Isolation and identification of IPU degrading bacterial strain

**Fig. 1:**
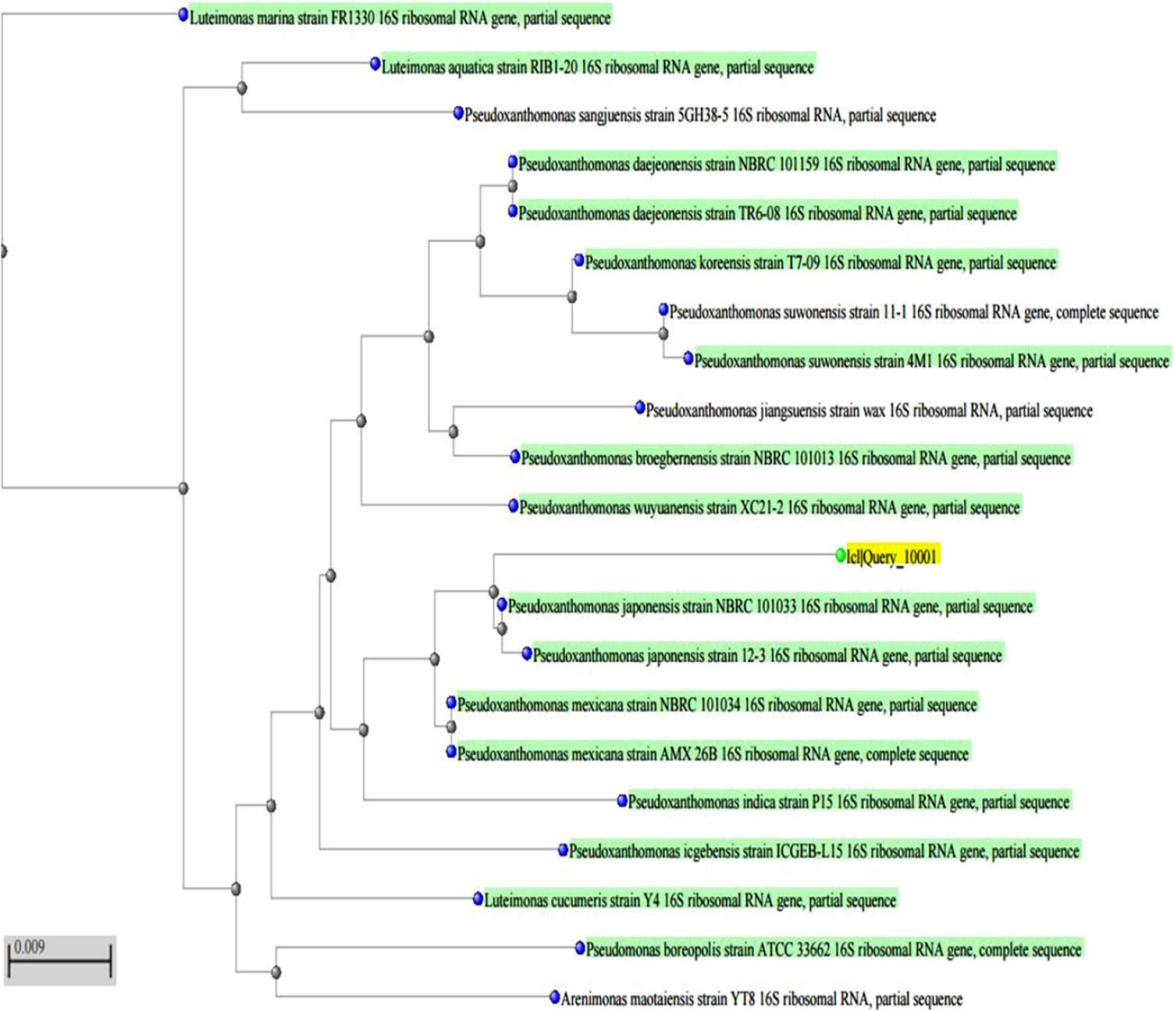
Phylogenetic tree based on MUSCLE multiple alignments computed for Mole-BLAST

Enrichment culture method was used to isolate IPU degrading bacterial isolate from herbicide-applied wheat fields. The isolate was small rod-shaped, gram-negative, aerobic, non-spore forming bacterium and designated as K2. K2 was tested for substrates utilization (24 carbohydrates as carbon source and 2 enzymes, viz. oxidase and catalase) to study its metabolizing abilities using KB009 part A and B HiCarbohydrate™ Kit. These tests were based on the principle of substrate utilization and pH change. The isolate showed positive reaction for oxidase, catalase and 20 carbohydrates (Table 1). 16S rRNA partial gene sequence analysis was used to identify the bacterial isolate. Homology search using BLAST revealed 95 % congruence of this sequence with 16S rRNA gene sequence of *Pseudoxanthomonas japonensis* (GenBank Accession No. NBRC101033). Phylogenetic tree constructed using Mole-Blast showed close relationship of this bacterial isolate (Genbank accession number KF279695) with *Pseudoxanthomonas japonensis* isolates (Fig. 1), Based on the bacterial growth on mineral salt agar plates supplemented with IPU as a source of carbon at different concentrations, 350 mg l^−1^ IPU was recorded as the minimum inhibitory concentration for its growth.

**Table 1:** Biochemical characteristics of IPU degrading bacterial isolate

Part A (KB009)	Result	Part B (KB009)	Results
Lactose	**+**	Inulin	**+**
Xylose	**+**	Sodium gluconate	**−**
Maltose	**+**	Glycerol	**−**
Fructose	**+**	Salicin	**+**
Dextrose	**+**	Glucosamine	**+**
Galactose	**+**	Dulcitol	**+**
Raffinose	**+**	Inositol	**+**
Trehalose	**+**	Sorbitol	**+**
Melibiose	**+**	Mannitol	**+**
Sucrose	**+**	Adonitol	**+**
L-Arabinose	**−**	α-Methyl-D-Glucoside	**+**
Mannose	**+**	Ribose	**−**
Catalase	**+**	Oxidase	**+**

+ positive, − negative test

### Effect of pH and temperature on biodegradation of IPU

The effect of pH and temperature on the IPU degrading potential of K2 was studied for 20 days. Both the factors showed strong effect on IPU degradation kinetics of K2 in broth medium. Initially IPU degradation was slow in all the pH and temperature ranges. After 10 days of incubation, it was increased significantly. IPU degradation by K2 at 25 °C and pH 6.5, 7.0, and 7.5 was 139.34 ± 0.20, 146.68 ± 0.60 and 143.19 ± 0.37 mg l^−1^, respectively (Table 2). After 20 days of incubation, maximum, i.e., 165.73 ± 0.59 mg l^−1^ IPU degradation was observed at pH 7.0 and 30 °C. At pH 7.5 and 6.5, degradation was 154.89 ± 0.51 and 153.13 ± 0.36 mg l^−1^, respectively (Table 3). However, IPU degradation at 35 °C and pH 6.5, 7.0, and 7.5 was 146.25 ± 0.22, 157.63 ± 0.39 and 149.85 ± 0.53 mg l^−1^, respectively (Table 4). Biodegradation of IPU in control flasks was far less than the inoculated flasks. IPU degradation by K2 at all the temperatures and pH values varied significantly (*P* < 0.05) with maximum degradation at neutral pH and 30 °C.

**Table 2:** Biodegradation of isoproturon by *Pseudoxanthomonas* sp. at 25 °C and different pH

Time (days)	pH 6.5		pH 7.0		pH 7.5	
	Control	Treatment	Control	Treatment	Control	Treatment
5	3.71 ± 0.20 (1.86)	13.16 ± 0.25 (6.58)	6.00 ± 0.38 (3.0)	21.08 ± 0.28 (10.54)	4.48 ± 0.30 (2.24)	17.93 ± 0.53 (8.97)
10	6.07 ± 0.26 (3.04)	46.57 ± 0.60 (23.29)	11.01 ± 0.44 (5.51)	61.28 ± 0.68 (30.64)	7.21 ± 0.17 (3.61)	55.17 ± 0.46 (27.59)
15	9.31 ± 0.34 (4.66)	97.72 ± 1.14 (48.86)	18.21 ± 0.28 (9.11)	118.78 ± 0.35 (59.39)	11.93 ± 0.47 (5.97)	113.19 ± 0.59 (56.6)
20	16.00 ± 0.25 (8.0)	139.34 ± 0.20 (69.67)	20.02 ± 0.73 (11.01)	146.68 ± 0.60 (73.34)	17.70 ± 0.52 (8.85)	143.19 ± 0.37 (71.6)

	SEm±	CD at 5 %	SEm±	CD at 5 %	SEm±	CD at 5 %
A (control)^ns^	0.25	0.76	0.25	0.75	0.22	0.67
B (treatment)**	0.35	1.07	0.35	1.06	0.31	0.95
A*B (interaction)**	0.50	1.52	0.51	1.50	0.44	1.35

Values are degraded amount of IUP in mg l^−1^, initial IPU concentration = 200 mg l^−1^, ± SE, *n* = 3

Values in parenthesis represent % IPU degradation

Control: uninoculated, Treatment: inoculated with *Pseudoxanthomonas* sp.

**  Significantly different from the control at *p* < 0.05

**Table 3:** Biodegradation of isoproturon by *Pseudoxanthomonas* sp. at 30 °C and different pH

Time (days)	pH 6.5		pH 7.0		pH 7.5	
	Control	Treatment	Control	Treatment	Control	Treatment
5	4.71 ± 0.20 (2.36)	27.78 ± 0.11 (13.89)	8.04 ± 0.37 (4.0)	35.34 ± 0.42 (17.67)	6.82 ± 0.63 (3.41)	30.01 ± 0.58 (15.01)
10	8.40 ± 0.38 (4.2)	48.14 ± 0.19 (24.07)	13.34 ± 0.34 (6.67)	59.70 ± 0.54 (29.85)	10.21 ± 0.72 (5.11)	52.14 ± 0.75 (26.07)
15	11.65 ± 0.64 (5.83)	115.04 ± 0.81 (57.52)	19.88 ± 027 (9.94)	134.67 ± 0.37 (67.34)	15.60 ± 0.70 (7.80)	121.62 ± 0.63 (60.81)
20	17.02 ± 0.25 (9.0)	153.13 ± 0.36 (76.57)	22.02 ± 0.86 (11.51)	165.73 ± 0.59 (82.87)	18.70 ± 0.58 (9.35)	154.89 ± 0.51 (77.45)

	SEm±	CD at 5 %	SEm±	CD at 5 %	SEm±	CD at 5 %
A (control)^ns^	0.21	0.65	0.25	0.75	0.32	0.97
B (treatment)**	0.30	0.92	0.35	1.07	0.45	1.37
A*B (interaction)**	0.43	1.30	0.50	1.51	0.64	1.94

Values are degraded amount of IUP in mg l^−1^, initial IPU concentration = 200 mg l^−1,^ ± SE, *n* = 3

Values in parenthesis represent % IPU degradation

Control: uninoculated, treatment: inoculated with *Pseudoxanthomonas* sp.

**  Significantly different from the control at *p* < 0.05

**Table 4:** Biodegradation of isoproturon by *Pseudoxanthomonas* sp. at 35 °C and at different pH

Time (days)	pH 6.5		pH 7.0		pH 7.5	
	Control	Treatment	Control	Treatment	Control	Treatment
5	3.65 ± 0.33 (1.83)	23.62 ± 0.33 (11.81)	6.29 ± 043 (3.15)	29.43 ± 0.71 (14.72)	5.48 ± 0.40 (2.74)	25.78 ± 0.54 (12.89)
10	5.40 ± 0.21(2.70)	48.35 ± 0.97 (24.18)	10.34 ± 0.34 (5.17)	49.53 ± 0.75 (24.77)	6.88 ± 0.16 (3.44)	43.05 ± 0.59 (21.53)
15	9.98 ± 0.39 (4.99)	114.78 ± 0.54 (57.39)	16.21 ± 0.82 (8.11)	123.73 ± 0.71 (61.87)	11.61 ± 0.70 (5.8)	118.31 ± 0.89 (59.16)
20	17.33 ± 0.17 (8.67)	146.25 ± 0.22 (73.13)	19.02 ± 0.15 (9.51)	157.63 ± 0.39 (78.82)	16.70 ± 0.51 (8.35)	149.85 ± 0.53 (74.93)

	SEm±	CD at 5 %	SEm±	CD at 5 %	SEm±	CD at 5 %
A (control)^ns^	0.23	0.69	0.29	0.87	0.29	0.87
B (treatment)**	0.32	0.98	0.41	1.24	0.48	1.23
A*B (interaction)**	0.46	1.39	0.58	1.75	0.56	1.74

Values are degraded amount of IUP in mg l^−1^, initial IPU concentration = 200 mg l^−1^ ± SE, *n* = 3

Values in parenthesis represent % IPU degradation

Control: uninoculated, treatment: inoculated with *Pseudoxanthomonas* sp.

**  Significantly different from the control at *p* < 0.05

The cumulative effect of pH and temperature on IPU biodegradation was assessed under laboratory conditions and maximum biodegradation of IPU was observed at 30 °C and 7.0 pH followed by 35 °C and pH 7.5. However, least degradation was observed at 25 °C and pH 6.5. IPU biodegradation was increased with increasing incubation time (Tables 2, 3, 4). Two factorial CRD analysis of experimental data revealed that IPU degradation by K2 varied significantly (*P* < 0.05) in all the pH and temperature regimes as compared to the uninoculated control. IPU degradation at pH 7.0 and 30 °C varied significantly (*P* < 0.05) than that of pH 6.5 and 7.5, but no significant difference in the IPU degradation was observed at pH 6.5 and 7.5. Similarly, IPU degradation at 35 and 25 °C varied very less with no significant difference. Statistical analysis confirmed that degradation of IPU was strongly influenced by pH and temperature. IPU degradation at pH 7.0 and 30 °C was significantly (*P* < 0.05) higher as compared to other pH and temperatures. Therefore, pH 7.0 and 30 °C were observed as the optimum conditions for the IPU degrading capacity of K2. 4-isopropylaniline was detected as degradation by-product in the medium (Table 5).

**Table 5:** Concentration of 4-isopropylaniline accumulated in the medium

Time (days)	pH 6.5		pH 7.0		pH 7.5	
	Control	Treatment	Control	Treatment	Control	Treatment
Temperature 25 °C						
5	ND	0. 60 ± 0.02	ND	0.87 ± 0.20	ND	0.98 ± 0.30
10	ND	2.01 ± 0.04	ND	2.34 ± 0.04	ND	1.67 ± 0.32
15	ND	3.34 ± 0.70	ND	3.97 ± 0.23	ND	2.13 ± 0.23
20	ND	3.91 ± 0.31	ND	4.01 ± 0.52	1.08 ± 0.05	2. 76 ± 1.02
Temperature 30 °C						
5	ND	0.78 ± 0.21	ND	1.88 ± 0.64	ND	2.47 ± 0.31
10	ND	2.66 ± 0.11	ND	3.77 ± 0.17	ND	3.45 ± 1.03
15	0.67 ± 0.02	3.56 ± 0.07	0.53 ± 0.02	4.23 ± 0.42	1.05 ± 0.10	3.89 ± 0.23
20	0.91 ± 0.06	4.22 ± 0.12	1.51 ± 0. 40	4.78 ± 0.12	1.79 ± 0.06	4.11 ± 0.10
Temperature 35 °C						
5	ND	1.23 ± 0.05	ND	1.54 ± 0.32	ND	1.26 ± 0.17
10	ND	2.18 ± 0.21	ND	1.96 ± 0.16	0.67 ± 0.14	2.47 ± 0.10
15	0.89 ± 0.12	2.87 ± 0.51	1.50 ± 0.21	2.86 ± 1.20	1.58 ± 0.23	3.56 ± 0.26
20	1.68 ± 0.31	3.14 ± 0.13	1.74 ± 0.07	3.54 ± 0.32	1.88 ± 0.21	3.86 ± 0.17

Values are presented in mg l^−1^, *ND* not detected, ±SE, *n* = 3

Biodegradation of IPU has already been studied in several soils repeatedly treated with IPU (Sorensen et al. 2001; Bending et al. 2003; El-Sebai et al. 2005, 2007). Bacterial metabolism of IPU and related phenylurea herbicides is one of the most reliable, cost effective and eco-friendly methods for reducing environmental burdens (Pieuchot et al. 1996; Hussain et al. 2011). Since several bacteria have been reported to have the phenylurea herbicides degrading ability (Dejonghe et al. 2003; El-Sebai et al. 2004; Sorensen et al. 2001; Widehem et al. 2002), the previous reports together with the present study indicate that different strains belonging to different genera can metabolize IPU. Interestingly, the strains differed in their characteristics of degradation capabilities (Sun et al. 2009). Pesticide degradation potential of the soil micro-flora is greatly subjected to pedoclimatic and physico-chemical conditions of the contaminated environment (Smith et al. 1997; Andrea et al. 2000). Moreover, pH and temperature significantly influence the pesticide-degrading capabilities of microorganisms (Bending et al. 2001, 2003; Walker et al. 2001; Rasmussen et al. 2005). Although the effect of pH on IPU degradation has also been reported in previous studies (Bending et al. 2003; Hussain et al. 2009, 2011; Sun et al. 2009), but the mechanisms responsible for this regulation have not been explained yet (Sun et al. 2009). The pH is known to affect the growth and survival of microbial populations (Russell and Dombrowski 1980; Sun et al. 1998; Rousk et al. 2009), which ultimately affects their physiological and metabolic activities. The results of the present study indicate that K2 have the ability to degrade significant quantities of IPU at pH range of 7.0–7.5 with an optimum activity at pH 7.0 and reduced activity at pH 6.5. This supports the fact that pH has an effect on the growth or survival of the bacterial population, which explains the variation in IPU degradation rate observed at different pH values The data recorded over the incubation time revealed that during the first 5–10 days, there was slow biodegradation of IPU, which might represent a lag phase while it got accelerated as the incubation proceeded, most likely due to induction/activation of enzymes in the inoculated cultures (Hussain et al. 2007; Giri et al. 2014). In the present investigation along with pH, temperature has also been observed as a key factor for IPU degradation by K2. Experimental data revealed maximum IPU degradation in 30 °C. It is very likely that 30 °C might be more conducive to bacterial growth than other incubation temperatures (Hussain et al. 2007).

Temperature plays a key role in bacterial metabolism and slight variation in temperature may increase or decrease the metabolic rates of the microorganisms. Interestingly, among the previously reported metabolites of IPU biodegradation, only 4-isopropylaniline was detected in the medium. The absence of other reported by-products may either be due to their formation below detection limits or simultaneous utilization by the bacterium as a carbon source (Weir et al. 2006).

### Effect of supplementary carbon on IPU degradation

**Fig. 2:**
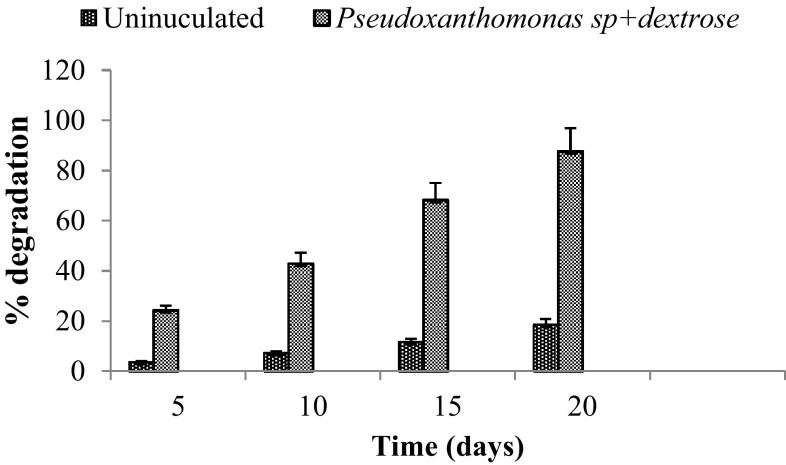
Biodegradation of isoproturon in the presence of auxiliary carbon source (*error bar *shows ± SE, *n* = 3)

Biodegradation of IPU in the presence of auxiliary carbon source was enhanced significantly as compared to the degradation as sole carbon source. At the end of 5 days, 3.63 % IPU was disappeared from the uninoculated flasks while 24.24 % IPU was utilized by K2. Initially, the degradation of IPU was slow, but it was accelerated between 15 and 20 days. After 20 days of incubation, maximum 87. 59 % (183.19 ± 0.94 mg l^−1^) IPU was mineralized by K2. Statistical analysis confirmed that IPU degradation in both the treatments varied significantly (*P* < 0.05) (Fig. 2). Significant increase in IPU degradation efficiency was observed in cometabolism, where dextrose was added as a supplementary carbon source. About 4.72 % enhancement in IPU degradation was observed due to supplementary carbon source at pH 7.0 and 30 °C as compared to the treatment without dextrose. 4-isopropylaniline was accumulated as by-product of the IPU degradation in the medium (Table 6).

**Table 6:** Concentration of 4-isopropylaniline accumulated in the medium

Time (days)	Control	*Pseudoxanthomonas* sp. + dextrose
5	ND	0.78 ± 0.21
10	ND	1.65 ± 0.02
15	ND	3.76 ± 0.33
20	2.07 ± 0.56	4.03 ± 0.27

Values are given in mg l^−1^, *ND* not detected, ± SE, *n* = 3

Addition of auxiliary nutrients stimulates microbial population in the pesticide polluted environment and enhances their catabolic capacities (Scow and Hicks 2005). Comprehensively, biostimulation involves the introduction of adequate amount of nutrients, and oxygen in the medium, to enhance pesticide degradation activity of microbes (Couto et al. 2010) or to promote co-metabolism (De Lorenzo 2008). The concept of biostimulation is to boost the intrinsic degradation potential of a soil micro-flora through the accumulation of amendments, nutrients, or other limiting factors, and has been used for the degradation of wide array of xenobiotics (Kadian et al. 2008). Addition of auxiliary carbon to the system having xenobiotic compounds increased the IPU biodegradation potential of bacterial cultures which is often because of increase in metabolic activities of the microbes. Kumar and Philip (2006a) also reported significant increase in endosulfan degradation in the presence of dextrose as a co-metabolic reaction in the medium. This particular fact can be attributed to IPU degradation in the present investigation. However, some studies have reported that the addition of auxiliary carbon to the system declined the degradation of pesticide compounds (Awasthi et al. 1997; Kumar and Philip 2006b; Goswami and Singh 2009). This might be because dextrose being an easily available carbon source as compared to pesticide and therefore the bacteria preferred dextrose over xenobiotics as an energy source. The study indicated that during the first 5–10 days, IPU degradation was slow, which can be attributed to a lag phase growth phase. However, it accelerated as the incubation proceeded, most likely due to induction/activation of catabolic enzymes in the inoculated medium (Giri and Rai 2012).

The study established correlation between pH, temperature, effect of auxiliary carbon source and IPU mineralization performance of K2 under laboratory conditions. These conditions vary from place to place in natural environment; therefore optimization of such factors is crucial while studying the biodegradation of pesticides. The results of this study entail that the enriched soil bacterial isolate possess efficient catalytic capabilities for IPU degradation. Therefore, this bacterial isolate may be useful for bioremediation of pesticide contaminated soil and water environment. However, further research will be needed to study the involvement of different catabolic genes and enzymes in each step of IPU mineralization and its implication in microcosm and field-scale bioremediation studies.
